# Does the operations of the National Health Insurance Scheme (NHIS) in Ghana align with the goals of Primary Health Care? Perspectives of key stakeholders in northern Ghana

**DOI:** 10.1186/s12914-016-0096-9

**Published:** 2016-08-30

**Authors:** John Koku Awoonor-Williams, Paulina Tindana, Philip Ayizem Dalinjong, Harry Nartey, James Akazili

**Affiliations:** 1 Regional Health Directorate, Ghana Health Service PMB, Upper East Region, Bolgatanga, Ghana; 2 Regional Health Directorate, Ghana Health Service, PMB, Bolgatanga, Upper East Region Ghana; 3 Navrongo Health Research Centre, Ghana Health Service, P. O. Box 114, Navrongo, Ghana

**Keywords:** Misalignment, Universal Health Coverage, National Health Insurance, Primary health care, Upper East region, Ghana

## Abstract

**Background:**

In 2005, the World Health Assembly (WHA) of the World Health Organization (WHO) urged member states to aim at achieving affordable universal coverage and access to key promotive, preventive, curative, rehabilitative and palliative health interventions for all their citizens on the basis of equity and solidarity. Since then, some African countries, including Ghana, have taken steps to introduce national health insurance reforms as one of the key strategies towards achieving universal health coverage (UHC). The aim of this study was to get a better understanding of how Ghana’s health insurance institutions interact with stakeholders and other health sector programmes in promoting primary health care (PHC). Specifically, the study identified the key areas of misalignment between the operations of the NHIS and that of PHC.

**Methods:**

Using qualitative and survey methods, this study involved interviews with various stakeholders in six selected districts in the Upper East region of Ghana. The key stakeholders included the National Health Insurance Authority (NHIA), district coordinators of the National Health Insurance Schemes (NHIS), the Ghana Health Service (GHS) and District Health Management Teams (DHMTs) who supervise the district hospitals, health centers/clinics and the Community-based Health and Planning Services (CHPS) compounds as well as other public and private PHC providers.

A stakeholders’ workshop was organized to validate the preliminary results which provided a platform for stakeholders to deliberate on the key areas of misalignment especially, and to elicit additional information, ideas and responses, comments and recommendations from participants for the achievement of the goals of UHC and PHC.

**Results:**

The key areas of misalignments identified during this pilot study included: delays in reimbursements of claims for services provided by health care providers, which serves as a disincentive for service providers to support the NHIS, inadequate coordination among stakeholders in PHC delivery; and inadequate funding for PHC, particularly on preventive and promotive services. Other areas are: the bypassing of PHC facilities due to lack of basic services at the PHC level such as laboratory services, as well as proximity to the district hospitals; and finally the lack of clear understanding of the national policy on PHC.

**Conclusion:**

This study suggests that despite the progress that has been made since the establishment of the NHIS in Ghana, there are still huge gaps that need urgent attention to ensure that the goals of UHC and PHC are met. The key areas of misalignment identified in this study, particularly on the delays in reimbursements need to be taken seriously. It is also important for more dialogue between the NHIA and service providers to address key concerns in the implementation of the NHIS which is key to achieving UHC.

## Background

In 2005, the World Health Assembly (WHA) of the World Health Organization (WHO) urged member states to aim at achieving affordable universal coverage and access to key promotive, preventive, curative, rehabilitative and palliative health interventions for all their citizens on the basis of equity and solidarity [1]. And then in December 2012 the United Nations General Assembly adopted a landmark resolution on Universal Health Coverage (UHC) [2]. UHC comprises of three dimensions; the breadth, depth and height of health service coverage. These dimensions take into account the proportion of population covered, the range of health services covered and the proportion of health costs covered. Essentially, UHC ensures that all populations have access to the required health services without anyone facing financial difficulty in paying for these services [2]. This can be achieved through the introduction of risk pooling and prepayment schemes like health insurance programmes. Governments around the world are taking action following these declarations; China, Thailand, South Africa, and Mexico are among the emerging economies that are rapidly scaling up public investment in health. Many low-income countries, especially in Africa, have also introduced health insurance reforms as a first step towards achieving UHC [3–6].

Although questions still remain about the impact of these health care reforms in improving access to primary health care (PHC), many countries continue to strive for UHC. In a review on the impact of UHC schemes in developing countries, Giedion et al. reported that UHC generally improves access to health care and to some extent financial protection [4]. However, their report also highlighted huge gaps. Meeting the goals of UHC requires that the necessary health systems are in place to promote access to healthcare. Thus it is important for UHC to be aligned to the goals of PHC which seeks to promote access to basic health care for all regardless of race, religious/political affiliation, gender, socio-economic status, location, etc.

### The Ghana National Health Insurance Scheme (NHIS)

In 2003, the government of Ghana established the National Health Insurance Scheme through an act of parliament (Act 650, revised to Act 852 in 2012) to provide financial risk protection against the cost of basic health care for all residents in Ghana. This new initiative was to replace the ‘cash and carry system’, an out of pocket payment system which limited the ability of many Ghanaians to access quality health care in the country. By law, all Ghanaians are required to join the NHIS, with the exception of the Ghana Armed Forces and the Ghana Police Service. A unique feature of the NHIS is that it incorporates both the formal and informal sectors. The formal sector makes a monthly payment of 2.5 % (direct deduction) out of their contributions to the Social Security and National Insurance Trust (SSNIT) to the NHIS. On the other hand, the informal sector makes direct payments of premium to the NHIS. Apart from the premiums from the formal and informal sectors, the NHIS is also financed through a levy of 2.5 % on goods and services (National Health Insurance levy), donor funding, Government of Ghana funding, and profits from investments made by the NHIA.

However, the NHIS makes provision for the exemption of certain categories of persons. These categories comprises of the indigent (poor and vulnerable), children who are below the ages of 18 years, the elderly who are above 70 years of age, pensioners of SSNIT, pregnant women and of late, persons with mental disorders.

The benefit package for the NHIS covers about 95 % of the diseases affecting Ghanaians. For curative conditions, there is no limit on the payment of medical bills for conditions covered under the benefit package. In addition, there are no co-payments, co-insurance or deductibles on the part of clients of the NHIS. As at the end of December, 2013, enrolment by Ghanaians to the NHIS stood at 36.8 % (MOH 2014). Though joining the NHIS in theory is mandatory, in practice the law is difficult to be enforced because the population is still largely informal. Studies on enrolment into the NHIS have demonstrated that premium payment hinders registration into the NHIS, especially for the informal sector [7, 8]. This may partly explain the state of the current national coverage figure for the NHIS.

While Ghana’s NHIS has been praised globally as an excellent example of how UHC can be promoted and implemented in Low and Middle-income countries (LMICs), the system has not been without challenges [9, 10]. Since 2014 for example, a number of private and faith-based health care providers have threatened to withdraw health services provided to clients of the NHIS due to lack of reimbursement from the NHIA. This calls for a holistic assessment of the opportunities/challenges present in the operations of the NHIS, particularly for PHC service delivery. Primary health care providers in the study settings include both public and private district hospitals, health centres/clinics, and the CHPS compounds. These health facilities are mandated to carry out health service delivery to populations within their catchment areas. They are also required to make referral for cases beyond their capacity.

However, there is limited evidence on the linkage between the operations of the NHIS and PHC service delivery. The aim of this study therefore was to identity key areas of misalignment between the operations of the NHIS with PHC and to suggest ways of addressing existing challenges. This was done by exploring how the NHIS interacted with stakeholders and other health sector programmes to promote the goals of UHC and PHC.

### Brief overview of Ghana’s health care system

**Fig. 1:**
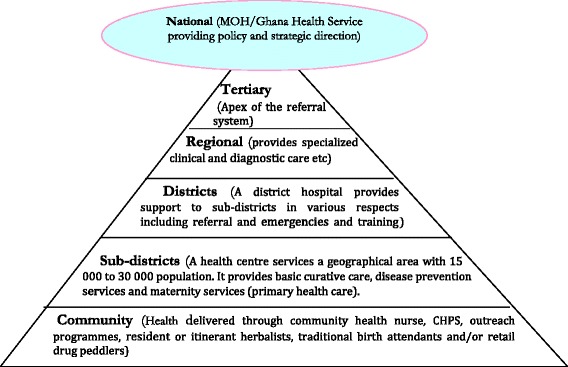
Organizational structure of Ghana health services delivery

Ghana’s health care system is currently managed by the Ministry of Health (MOH), which provides overall policy direction [11]. But the operation of the policy is the responsibility of the Ghana Health Service (GHS). Functionally, the GHS is organized at five Levels (see Fig. 1).

At the apex are the tertiary hospitals which are also teaching hospitals. This is followed by the regional hospitals that provide specialist care and are reference points for the district hospitals. The district level facilities provide care at the district level and also serve as referral centres for the sub-district facilities. The lowest facilities in the health delivery ladder are the community clinics. A relatively unique aspect of the public health system in Ghana is the Community-based Health Planning and Services (CHPS). Operationally, CHPS is defined as a “strategy for the health care delivery system to provide cost-effective and adequate quality basic primary health services to individuals and households in the communities where they live through engaging the community in the planning and delivery of services” [12]. CHPS basically entails a nurse living in a defined community and offering limited curative and preventive health care services. Even though CHPS is seen as a well-tested strategy for reducing inequities and promoting geographical access to basic health care, its main challenge is the enormous resources required to roll out the strategy to all parts of the country. Therefore, if the operations of the NHIS is well defined and aligned with the goals of PHC, it is likely to support the provision of curative, as well as promotive and preventive interventions needed to improve the health of Ghanaians.

### The Joint Learning Network

The Joint Learning Network (JLN) is an international network of health practitioners and policy makers including representatives from Ghana. The network recently focused on promoting alignments between the goals of UHC and PHC [13]. The term “alignment” as used by the network refers to the ‘identification of ways in which health financing authorities can better interact with, support, or incentivize PHC actions and goals, through monetary and non-monetary means. One of the key areas of interest to the network is getting a better understanding of how member countries’ health insurance schemes or financial coverage institutions interact with stakeholders and other health sector programmes in promoting the goals of PHC. To achieve this, the network developed a tool to help the various countries to document and self-assess the alignments and misalignments between the goals of UHC and PHC.

## Methods

The study involved a survey and qualitative semi-structured interviews with key stakeholders in six selected districts in the Upper East region of Ghana.

### Research setting

The Upper East region is one of the 10 regions of Ghana. With an estimated population of 1,046,545, the region is divided into 13 districts. The region has one regional hospital located in Bolgatanga, the regional capital; 5 district hospitals; 26 health centres and 35 clinics run by the Ghana Health Service, mission institutions and the private sector. Each district is managed by a District Health Management Team (DHMT) which supervises about 212 health centres and CHPS compounds [14].

### Sampling

Six (6) out of the thirteen (13) districts in the region were purposively selected for the study. Within each of the districts that were selected, we mapped the relevant stakeholders responsible for facilitating access to PHC services. These included the regional health directorate and district health management teams, district health insurance schemes, as well as both private and public PHC providers. We also selected the district mutual health insurance schemes from each of these selected districts for interview.

### Survey Tools

The study adapted the survey tools developed by the JLN PHC initiative [15], meant to document the alignments as well as misalignments between the goals of UHC and PHC. The survey tools comprised of five modules targeting various categories of stakeholders including the Ministry of Health, Ministry of Finance, the Health Insurance Institutions and PHC providers. Each of these modules sought to obtain data on: a. priority setting in the country’s or state’s health policy agenda; b. financing policies (i.e., revenue generation); c. payment policies—determining what to pay for and how to pay providers; d. influencing the population’s and providers’ actions through regulations and communications; and e. monitoring and evaluation (including data sharing) [15].

Thus the study in the Upper East region of northern Ghana focused on exploring the misalignments of the NHIS at the regional and district levels of health service organization and delivery. The target respondents included the regional coordinator of the NHIA and the district coordinators of the NHIS in the 6 sampled districts. The tool for the MOH was administered to the Ghana Health Service and the main respondents were the regional director of health services and the district directors in DHMTs who are responsible for the supervision of the district hospitals, health centers/clinics and CHPS compounds.

### Qualitative interviews

This study included qualitative face to face semi-structured in-depth interviews with all the stakeholders targeted for this study and focus group discussions in some cases. In each selected institution, staff who were familiar with the inner workings of the institution in terms of its financial health coverage and operations were specifically targeted. In most cases, the interviews were conducted with the medical directors of the facilities, while in others the interviews were conducted with the accountants and administrators.

### Stakeholders’ workshop

Following the completion of the survey and qualitative interviews, a stakeholders’ workshop was held to validate the preliminary results, to provide a platform for stakeholders to deliberate on the key areas of misalignment between the NHIS and PHC. The workshop was also meant to elicit additional information, ideas and responses from participants as well as provide recommendations for achieving the goals of UHC and PHC. Participants included all the stakeholders interviewed in the survey, facilitators of the NHIA, representatives from the regional health directorate and members of the research team.

The format of the workshop included presentations from the facilitators to provide more clarity on the JLN PHC tool, the national health insurance policy, and followed by general discussions. Smaller group discussions were further held to help identify and prioritize the key areas of misalignment and recommendations.

### Analysis of qualitative data

The qualitative interviews were conducted by two research assistants and a senior researcher. Majority of the respondents declined to be audio-recorded and extensive field notes had to be taken by the field personnel and typed into word. Interviews that were audio recorded were transcribed and included in the analysis.

Data analysis was on-going throughout the project, using a thematic approach [16, 17]. Each of the transcripts was reviewed independently by three researchers highlighting key themes emerging from the interviews. This was followed by a group discussion to review the data, compare the various themes and agree on key areas of misalignment based on the analysis of the data. Following the stakeholders’ workshop, the team reviewed the issues identified by the participants and ranked the key areas of misalignment specifically, based on both the results of the survey and the workshop.

### Analysis of quantitative data

Quantitative data were entered from the questionnaires into separate databases designed with Epidata 3.02 according to the different categories of the respondents. All data captured were verified and validated to ensure high data quality. Binary and categorical variables were summarised using percentages. For responses captured as percentages, the most frequent (mode) reported percentage, as well as the minimum and maximum responses were presented. Graphs were also plotted to display some tables where it was appropriate.

## Results

In all, 56 key stakeholders were interviewed including 30 men and 26 women. Some of the interviews with the PHC providers involved group discussions with the medical directors and accountants of the facilities as illustrated in the Table 1 below:

**Table 1:** Sample of stakeholders interviewed

Stakeholder group	Number of interviews
MOH/Ghana Health Service	7
National Health Insurance Scheme	6
Private health care providers	7
Public health care providers	33
Faith-based health care providers	3
Total	56

### Clarifying the concepts: Universal Health Coverage (UHC) and Primary Health Care (PHC)

Universal health coverage is a concept that is widely used and sometimes misunderstood. It seeks to promote the concept of universal access to health care and financial protection for all. PHC seeks to provide better health for all through the provision of quality and accessible health services. In this study a number of stakeholders suggested that it is important for these concepts to be clearly defined and understood by all actors:

*‘Let’s understand that UHC is a new term but it is being used mostly now to replace PHC. If you look at some of the literature, the linkage is that of course you need PHC to achieve UHC, not necessarily the opposite. When you talk of UHC you are talking about two things, availability of services and financial access, these are the two. You must have financial access but you don’t necessarily need financial access to attain some levels of PHC. From the perspective of the receiver of the services, if you have community health nurses who talk about health education, have a community durbar etc., everybody will have access to that information because they are in the community. It is when you are providing direct clinical services, you are treating malaria, you are providing immunization or family planning then people will have to pay some money then you are more or less saying that you cannot get that service if you don’t have financial access’ (IDI with Regional Manager, Ghana Health Service).*

Thus, PHC was generally referred to as a gatekeeper system by most respondents. As supported by the quotes below, most of the stakeholders explained that clients are often advised to seek care at the PHC level, particularly the health centers in the community as the first point of call before proceeding to higher facilities through a referral system.

*‘Normally we encourage all our clients to first of all know that when they are taken ill they should report at a place that they can access PHC so that if in that particular clinic or CHPS compound they are not able to treat the disease they will be referred to the hospital’ (IDI with DNHIS).*

*‘Yes, PHC is the gate-keeper system. Clients are encouraged to use the PHC first’ (IDI with DNHIS).*

### Current linkages between UHC and PHC

A number of respondents reported that there are some linkages between UHC and PHC. For example, a district director of health services suggested that the CHPS compounds provide health services to insured clients at the community and they are then reimbursed by the NHIS. Some health care providers were also of the view that the exemption policy for the poor, pregnant women and others within the NHIS is a good example of where the concept of UHC aligns well with PHC. There is therefore the need for all the actors (NHIA, MOH, GHS and the primary health care providers) to work together to ensure that these linkages are well coordinated.

The stakeholders from the MOH expressed their commitment to promote the concept of UHC within the region while the district coordinators of the NHIS also reported that they have a system for monitoring the activities of the PHC providers.

*‘We have a claims unit that goes round to the various providers and do attendance counts. So for them when you report at the facility that you are sick, there is a book there that they will enter your records; your name, your insurance number and everything. So at the end of the month we can tell how many people attended this particular facility as against facility B, may be facility A against B, against C and all those things. So looking at the numbers comparatively from month to month we know whether people are patronizing our gatekeeper system or not. So in doing so, we are always able to tell’ (IDI with DNHIS).*

Some of the private PHC providers confirmed that staff of the NHIS visits their facilities periodically to assess their operations.

*‘They monitor OPD books, folders and diagnosis books. They also inspect the laboratory and dispensary. Although this is not regular, they also look at issues on the waiting times of clients and the prescription practice of the facility’ (IDI with Private Service provider).*

There was also some evidence of a data sharing practice between the MOH and the NHIS:

*‘Yes, we use data from the NHIS to determine the health insurance coverage for the municipal/district/sub-district. Private providers operate under the auspices of Ghana Health Services. In fact, they are also trained by the GHS. Their data is used for monitoring and evaluation as well’ (IDI with DDHS).*

*‘We use our own data however, sometimes the MOH when they have their review meetings like midyear review meetings or end of year review meetings they do attend based on the information there’ (IDI with DNHIS).*

However, it was not very clear from stakeholders’ responses how this data is used by the various actors and whether these data actually inform the work of the MOH and NHIS.

### Sources of funding for primary health care

**Fig. 2:**
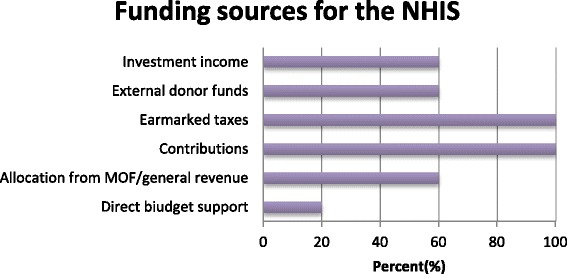
Funding sources for NHIS

In the interviews with the various stakeholders, most suggested that PHC is primarily funded by internally generated funds, funding from partners, NGOs, Member of Parliament’s health fund and contributions from the local communities in the form of providing labour for building infrastructure for CHPS compounds. Other sources include Government of Ghana budget allocations, as illustrated in the Fig. 2 below:

The public health service providers reported that the funds they receive from the NHIS are often used for purchasing drugs, paying supplementary staff, attending workshops and organizing outreach programmes.

*‘The monies from NHIS come in two forms, drugs (medicines) and non-drugs (services). We can’t use the drugs money. The non-drugs monies are used to run the facility such as fuel, outreaches, repairs and minor works on the facility’ (IDI with Public PHC provider).*

The NHIS funds are paid as reimbursements on a fee-for-service basis according to number of cases and type of cases seen by the facility on a fixed amount per service. In the case of public health service providers this money is used to pay for activities that the facility undertakes- merging with the other sources of funding for the facility. Though it can pay for supplementary staff - it is not used to pay monetary incentives to the staff. With the NHIS, incentives are not directly paid to service providers.

**Fig. 3:**
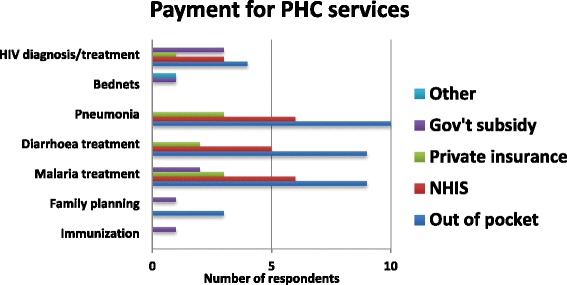
Payment for PHC services

Incentives are usually paid by Government of Ghana as part of the salaries to public sector as well as some private service providers, such as mission health facilities. This study did not address issues relating to incentives for service providers. Interestingly, despite the contribution of the NHIS to PHC services, the quantitative data suggests that many clients are still paying out of pocket for basic health services such as treatment and diagnosis for HIV, Malaria and Pneumonia, as Fig. 3 indicates. The exact figures for out of pocket expenditure were not collected for this study.

### Key impediments to achieving PHC objectives

**Fig. 4:**
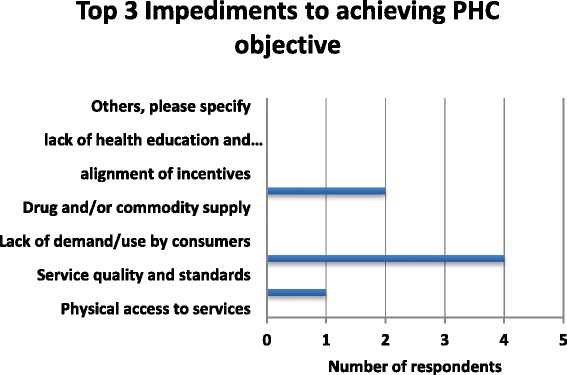
Top 3 Impediments to achieving PHC objectives

A number of stakeholders highlighted some of the key impediments to achieving PHC objectives to include the fact that the NIHS does not cover promotive and preventive services. As indicated in Fig. 4 below, the top three impediments that were highlighted in the study were insufficient funding, transportation barriers and human resource for health distribution.

**Fig. 5:**
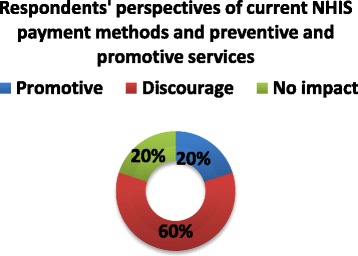
Current NHIS payment methods and their effect on the provision of preventive and promotive services

Most of the health service providers reported that delays in reimbursement for the services they render to clients has hugely affected their ability to meet PHC goals. About 50 % of the stakeholders interviewed were also dissatisfied with the speed at which these payments are often made. Approximately 60 % of the respondents reported that the current payment method used by the NHIS discourages the provision of preventive and promotive services at the PHC level, shown in Fig. 5. This is attributed to the fact the NHIS reimburses for only curative services that are covered under the benefit package at all levels of health care (primary secondary and tertiary).

The rationale for the non-payment of preventive services by the NHIS was attributed to the high cost of rendering such services. On the part of health facilities, they indicated that they lacked the requisite staff as well as adequate space to provide such services.

#### Bypassing PHC

Another major challenge highlighted in these interviews is the practice of bypassing the PHC level. Some of the service providers explained that some of the reasons for bypassing these facilities are the lack of some basic services at the PHC level such as laboratory services, as well as proximity to the district hospitals. As some service providers suggested:

*‘People have many choices these days so they will go to wherever they please. They will bypass to other facilities because of the treatment they will receive at that facility. Also, some laboratory equipment such as analyzer/HB machine is not in my facility but I can treat sepsis and anaemia well but because I don’t have this equipment they bypass my facility’ (IDI with Private PHC provider).*

*‘There are people who do facility shopping because of the NHIS’ (IDI with Public PHC provider).*

Another rationale for bypassing the PHC level has to do with clients’ uncertainty about the outcome of their ailment and the kind of support they will need. For example, some of the providers reported that pregnant women were more likely to bypass the PHC level because of uncertainties surrounding the delivery process:

*‘Because pregnant women are unsure of the outcomes of their labour during delivery so they go to the regional hospital where, if the need arises they could be operated upon’ (IDI with Public PHC provider).*

Most of the district hospitals complained that the trend of bypassing PHCs puts undue pressure on the services at the hospitals. They noted that the NHIS financial cover does not adequately take care of all aspects of primary health care. For instance, the NHIS does not cover health promotion and education and other preventive services. Similarly, other essential services including family planning, fuel and transport as well as cost of monitoring are not reimbursable. This block of services, many participants argued constitute a significant proportion of the service package of CHPS which largely defines Ghana’s primary health care strategy. The non-payment of these services by the NHIS therefore makes the scheme anti-primary health care, less supportive of the goals of universal health coverage.

Some participants were of the view that if the financial cover of Ghana’s NHIS included preventive services, it will significantly raise the private service providers’ contribution to the health sector quantum of care and hence contribute to improving the health status of the population. Currently, efforts to convince private service providers to add on some amount of preventive services to their service line have proven futile. Private service provider stakeholders who participated in the workshop strongly expressed the view that since their operations are largely based on cost recovery, and making profit to pay salaries of workers, purchase medical supplies, provide and maintain infrastructure, there is no incentive for providing preventive services if the resultant cost will not be borne by the system that finances health care services in the country.

Other lapses and misalignments attributed to the NHIS included the barring of health centers from prescribing some common medications, low tariffs and the difficulties people in rural communities in remote areas have with registering and renewing their NHIS cards.

### Key areas of misalignment between UHC and PHC

There was a general consensus among the various stakeholders, particularly from the MOH that the current focus of the NHIS on just curative services does not serve the purposes of promoting key principles underlining UHC and PHC. Most respondents suggested that the NHIS should include both promotive and preventive services such as family planning, health education and the CHPS services that will support the various service providers to provide these services. As the quotes below suggest:

*‘The NHIS does not pay for home visits made by CHOs, therefore staff are often reluctant to carry out these services which are crucial for PHC. Again, the non-payment of transportation cost by the NHIS does not promote the full utilization of health services. Insured clients are still unable to access health services because of transportation barriers. Furthermore, the non-payment by the NHIS for family planning services is another issue’ (Interview with DDHS).*

Some respondents also reported that the current mechanism for the disbursements of funds to the service providers is a big challenge. As one district director suggested:

*‘The NHIS usually makes payments to the Sub-districts which will then cater for the medicines and other supply needs of the various facilities under that sub-district. The practice is not good for the smooth operation of the providers especially the CHPS level’ (IDI with DDHS).*

In interviews with the NHIS, most of the respondents reported that although the scheme does not directly fund preventive and promotive health services, some of their activities involve public education on how to prevent diseases which indirectly supports disease prevention and promote healthy living. The cost of providing these services are usually taken up by the facilities themselves through their internally generated funds.

*‘We don’t have enough resources to do that because like you are saying if you are talking about sensitization sometimes when our PROs go on the radio station they sensitize people on these areas, for example, currently there is an advert running in the media concerning the health insurance authority. Those are the things that we do but to give you money that you should do this or that, that is what we don’t do. Recently I don’t know if you have been hearing on radio health insurance has sponsored an advert like that concerning Ebola, what you do to prevent you from getting Ebola and cholera. That is how far we can go but for us to give out money to an institution to do those things we don’t do’ (IDI with DNHIS).*

Some of the district NHIS coordinators further explained that PHC is a top priority, and in the accreditation process for service providers, one of the key criteria used is whether they provide preventive and promotive services as well. However, in practice it is clear that the emphasis is mainly on the curative services, as suggested by a district coordinator below:

*‘We don’t pay those things because they don’t submit claims on that, what they submit the claims on is disease condition. If you report ill at the clinic or the CHPS compound or the hospital, after your complain they will determine whether it is diarrhea, malaria or complicated malaria or hernia or anything, it is after that, that they will charge and bill us and at the end of the month we convert and pay but we don’t pay on preventive services’ (IDI with DNHIS).*

In both the survey and stakeholders’ workshop, participants highlighted the need for more collaboration between the various actors, particularly on identifying key areas of misalignments and creating opportunities for dialogue between the actors. A lot of emphasis was also placed on preventive and promotive services with calls for the NHIA to provide funding for these services. The workshop concluded with a call for a national primary health care policy and listed the following as top five areas of misalignment:

1. Delays in reimbursements of claims for services provided by healthcare providers. These delays are serving as a disincentive for service providers to embrace the NHIS.
2. Inadequate coordination among stakeholders in PHC delivery.
3. Inadequate funding for PHC, particularly on preventive and promotive services.
4. Bypassing PHC facilities due to lack of basic services at the PHC level such as laboratory services, as well as proximity to the district hospitals.
5. No national policy on primary health care in Ghana.

## Discussion

The WHO defines universal health coverage as all people having access to promotive, preventive, curative, rehabilitative and palliative health care services according to their need, which should be of sufficient quality, and that all clients of these services should not face financial hardship when paying for their usage [1]. This study suggests that there are on-going efforts to make the concept of UHC a reality in Ghana and the NHIS is a giant step towards achieving this objective. However, despite the success stories of the NIHS, there are serious challenges that need to be addressed.

### Key Impediments to achieving UHC

One of the key impediments to achieving UHC and PHC is the lack of financial resources and the human capacity to provide the necessary services at the PHC level. Empirical studies on the NHIS in Ghana have consistently documented dissatisfaction among health care providers with the undue delay in processing and payment for claims submitted [18, 19]. In this current study, while the NHIS lamented over the inadequacy of the funding they receive, the service providers also complained that the speed at which they are reimbursed for the services rendered to clients is unsatisfactory. This calls for the need for some priority setting within the NHIS. But the process of setting these priorities need to be informed through active engagement with the service providers who work closely with patients.

This study suggests that the benefit package offered within the current NHIS remains inadequate although it claims to be providing coverage of over 95 % of all disease burden in Ghana. One major concern arising is the exclusive focus of the current NHIS on curative services with limited emphasis on promotive and preventive services. As most of the service providers indicated, these services are mainly supported through their internally generated funds. They have therefore called for more financial support through the NHIS to intensify efforts at providing these services. The concern for the NHIS to include in its benefit package preventive and promotional health will be in the right direction to achieving UHC. After all UHC seeks complete access to quality health care without any barriers including financial.

### Reducing out of pocket payments

A key finding from this study is the report from the PHC providers that a high percentage of clients are still making out of payments for some basic PHC services, particularly in private PHC facilities. Although our study did not specifically set out to study this trend, similar findings have been recorded by other studies [20]. A recent study in two rural districts of Nkoranza and Offinso showed that individuals were still making out of pocket payments for health services, despite the implementation of the NHIS [21]. Although it is expected, the concern is why patients would choose to pay for services at private facilities, particularly if the NHIS is aimed at providing financial risk protection for patients. The finding calls to question whether there are issues about the quality of care provided as well as attitude of health providers. In a related study in the district [22], poor health staff attitudes were a major reason why some were not willing to join the NHIS. There are related studies from Senegal and Burkina Faso where quality of health care relating to staff attitudes is reported to be of concern to insured clients [23, 24]. Indeed further research may be required in this important area of health care delivery.

Our study has demonstrated that to achieve UHC, it is important for all key stakeholders such as the NHIA, the Ghana Health Service, Public and Private PHC providers and patient groups to work together to identify the key challenges and proposed solutions.

To promote UHC particularly at the PHC level, it is important to identify the activities of the PHC facilities and make sure that these are covered by the NHIS. The stakeholders have also suggested that the NHIS should include preventive services as a core component of the benefit package. This is in line with the principles underlying UHC to make preventive, promotive and curative services accessible and affordable to all [1].

## Conclusion

This study suggests that despite the progress that has been made since the establishment of the NHIS in Ghana, there are still huge gaps that need urgent attention to ensure that the goals of UHC and PHC are met. The key areas of misalignment identified in this study, particularly on the delays in reimbursements and the lack of coordination between the various stakeholders need to be taken seriously. It is also important for more dialogue between the NHIA and service providers to address key concerns in the implementation of the NHIS. Other key areas which require further research include reexamining the benefit package of the NHIS and the need to also address the issue of out of pocket payments within the NHIS. Addressing all these issues will make the achievement of the goals of UHC and PHC realizable.

## Abbreviations

CHC: Community Health Centres
CHPS: Community-based Health Planning and Services
DDHS: District Director of Health Services
DHMT: District Health Management Team
GHS: Ghana Health Service
JLN: Joint learning network
MOH: Ministry of health
NHIA: National Health Insurance Authority
NHIS: National Health Insurance Scheme
OOP: Out-of-pocket
PHC: Primary health care
UHC: Universal Health Coverage
WHA: World Health Assembly
WHO: World Health Organization
